# Supplementary material for: A Comprehensive Assessment of Ultraviolet-Radiation-Induced Mutations in Flammulina filiformis Using Whole-Genome Resequencing
Source: J Fungi (Basel). 2024 Mar 20;10(3):228. doi: 10.3390/jof10030228 (PMC10971301; doi:10.3390/jof10030228)
Supplement: Supplementary file 1 [file jof-10-00228-s001.zip › Supplementary Material S8/KEGG annotation/out/64381550635650.os/KO/out_map/map05110.html]

KEGG PATHWAY: Vibrio cholerae infection - Reference pathway


|  |  |
| --- | --- |
| **Vibrio cholerae infection - Reference pathway** |  |

[
Pathway menu
| Organism menu
| Pathway entry
| Show description
| User data mapping
]

Cholera toxin (CTX) is one of the main virulence factors of Vibrio cholerae. Once secreted, CTX B-chain (CTXB) binds to ganglioside GM1 on the surface of the host's cells. After binding takes place, the entire CTX complex is carried from plasma membrane (PM) to endoplasmic reticulum (ER). In the ER, the A-chain (CTXA) is recognized by protein disulfide isomerase (PDI), unfolded, and delivered to the membrane where the membrane-associated ER-oxidase, Ero1, oxidizes PDI to release the CTXA into the protein-conducting channel, Sec61. CTXA is then retro-translocated to the cytosol and induces water and electrolyte secretion by increasing cAMP levels via adenylate cyclase (AC) to exert toxicity.
Other than CTX, Vibrio cholerae generates several toxins that are perilous to eukaryotic cells. Zonula occludens toxin (ZOT) causes tight junction disruption through protein kinase C-dependent actin polymerization. RTX toxin (RtxA) causes actin depolymerization by covalently cross-linking actin monomers into dimers, trimers, and higher multimers. Vibrio cholerae cytolysin (VCC) is an important pore-forming toxin. The assembly of VCC anion channels in cells cause vacuolization and lysis.

|  |  |
| --- | --- |
| Reference pathway | 100% |
